# Diet-induced obesity links to ER positive breast cancer progression via LPA/PKD-1-CD36 signaling-mediated microvascular remodeling

**DOI:** 10.18632/oncotarget.15123

**Published:** 2017-02-06

**Authors:** Liuyi Dong, Ye Yuan, Cynthia Opansky, Yiliang Chen, Irene Aguilera-Barrantes, Shiyong Wu, Rong Yuan, Qi Cao, Yee Chung Cheng, Daisy Sahoo, Roy L. Silverstein, Bin Ren

**Affiliations:** ^1^ Blood Research Institute, Blood Center of Wisconsin, Milwaukee, Wisconsin, USA; ^2^ Edison Biotechnology Institute and Department of Chemistry and Biochemistry, Ohio University, Athens, Ohio, USA; ^3^ Department of Pathology, Medical College of Wisconsin, Milwaukee, Wisconsin, USA; ^4^ Diagnostic Radiology and Nuclear Medicine, University of Maryland Medical Center, Baltimore, Maryland, USA; ^5^ Department of Medicine, Medical College of Wisconsin, Milwaukee, Wisconsin, USA

**Keywords:** CD36, protein kinase D, phospholipid, lysophosphatidic acid, microvascular remodeling

## Abstract

Obesity increases cancer risk including breast cancer (BC). However, the direct regulatory mechanisms by which obesity promotes BC progression remain largely unknown. We show that lysophosphatidic acid/protein kinase D1 (LPA/PKD-1)-CD36 signaling is a *bona fide* breast cancer promoter via stimulating microvascular remodeling in chronic diet-induced obesity (DIO). We observed that the growth of an estrogen receptor (ER) positive breast cancer was markedly increased when compared to the lean control, and specifically accompanied by increased microvascular remodeling in a syngeneic BC model in female DIO mice. The tumor neovessels in DIO mice demonstrated elevated levels of alpha smooth muscle actin (α-SMA), vascular endothelial growth factor receptor 2 (VEGFR 2) and endothelial differentiation gene 2/LPA receptor1 (Edg2/LPA_1_), enhanced PKD-1 phosphorylation, and reduced CD36 expression. Tumor associated endothelial cells (TAECs) exposed to LPA demonstrated sustained nuclear PKD-1 phosphorylation, and elevated mRNA levels of ephrin B2, and reduced mRNA expression of CD36. TAEC proliferation also increased in response to LPA/PKD-1 signaling. These studies suggest that the LPA/PKD-1-CD36 signaling axis links DIO to malignant progression of BC via stimulation of *de novo* tumor arteriogenesis through arteriolar remodeling of microvasculature in the tumor microenvironment. Targeting this signaling axis could provide an additional novel therapeutic strategy.

## INTRODUCTION

Obesity contributes to the progression of breast cancer (BC) and other cancer types [1–3]. The causal mechanisms by which obesity is linked to BC risk and progression remain incompletely investigated [4, 5]. Moreover, the diverse scenarios of obesity lead to unexplainable contradictory results in the contributions of individual factors to obesity-related cancers [6, 7].

Obesity is known to stimulate angiogenesis [8], one of the hallmarks in the tumor microenvironment (TME) that promotes BC progression [2, 9, 10]. The questions that remain unanswered are whether or how signals from the stromal microenvironment in the obese state contribute to angiogenesis-mediated malignant progression of BCs and what type of proangiogenic process is associated with the BC progression. A seminal finding demonstrated that lysophosphatidic acid (LPA) signaling is essential for BC progression and is implicated in tumor angiogenesis in the TME [11, 12]. LPA as a lipid signaling mediator promotes microvascular remodeling and physiological angiogenesis [12–18] via activation of protein kinase D1 (PKD-1) signaling [14, 15], and is also implicated in the regulation of arteriogenesis [15, 19, 20]. In microvascular endothelial cells (MVECs), arteriogenic gene reprogramming is initiated once the transcription of *CD36* (an angiogenic regulator) is turned off in response to LPA/PKD-1 signaling, leading to proangiogenic and proarteriogenic responses [15, 21], whereas CD36 promotes glioblastoma progression [22] and metastatic potential of oral, breast and skin cancers in tumor initiating cells [23].

In obesity LPA is excessively produced in circulation due to increased expression of autotaxin in adipocytes [24], and plasma LPA levels are significantly elevated in mice with high-fat diet-induced obesity (DIO) [25, 26]. This led us to hypothesize that DIO-derived LPA contributes to BC progression by promoting angiogenesis via PKD-1 signaling. Using a syngeneic estrogen receptor (ER) positive (ER^+^) breast adenocarcinoma model in DIO mice and microvascular endothelial cells and tumor-associated endothelial cells (TAECs), we established that the LPA/PKD-1-CD36 signaling pathway may regulate microvascular remodeling through modulation of EC differentiation, thus linking obesity to the malignant progression of an ER-positive BC.

## RESULTS

### Diet-induced obesity promotes estrogen receptor positive breast cancer progression

**Figure 1:**
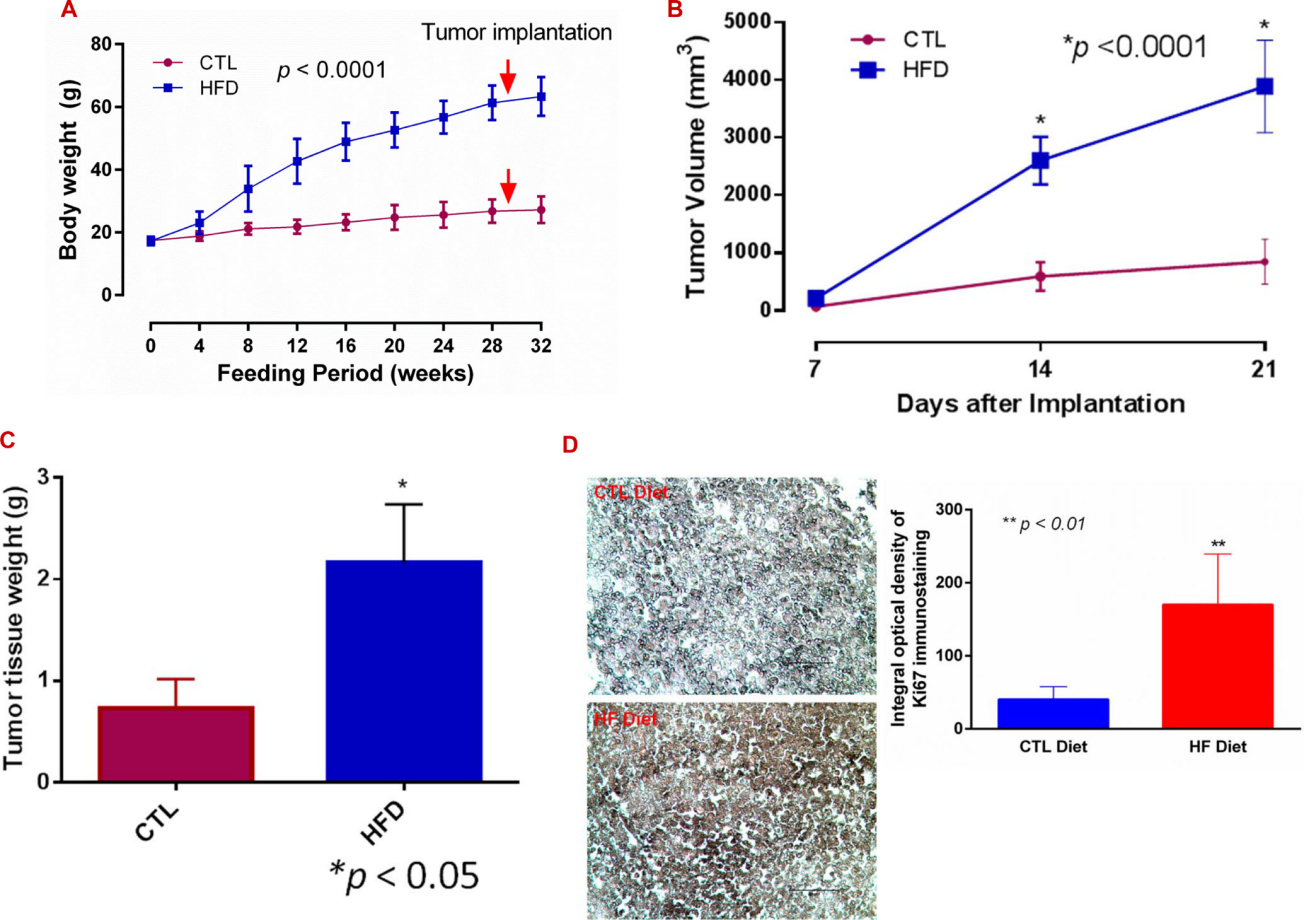
Diet-induced obesity promotes breast cancer progression (**A**) Female mice were maintained on a high fat (HF diet, *n* = 14) and on a control (CTL, *n* = 15) diet for the indicated time, and body weight was recorded. (**B**) E0771 cells were implanted subcutaneously close to the fourth mammary pad, and tumor volumes were measured and calculated. (**C**) Tumors were extracted and weighed at day 21 or earlier. (**D**) Tumor sections were stained with an anti-Ki67 antibody (Millipore) using VECTASTAIN^®^ABC with an alkaline phosphatase enzyme detection system. Images were acquired with a Nikon Eclipse E600 microscope, and Ki67 staining density was analysed with NIH Image J software. Bar = 50 μm in representative images.

Obesity reduced survival among women with ER^+^ breast tumors [27]. To determine the association of chronic DIO with BC progression, female mice were fed a high fat (HF) or control (CTL) diet for 32 weeks. Over the course of HF diet feeding, body weight steadily increased in the experimental group, showing a nearly three-fold increase compared with mice on a control chow diet (CTL) (Figure 1A, *p* < 0.0001). Consistent with a previous report [28], the DIO mice did not become hypercholesterolemic (Supplementary Figure 1A) but displayed elevated levels of plasma trigylcerides and fasting glucose (Supplementary Figure 1B and 1C). Non-fasting glucose was not changed significantly (Supplementary Figure 1D). The levels of plasminogen activator inhibitor 1 (PAI-1) and leptin in the plasma were increased relative to those in the control mice, but no significant differences were observed in the levels of plasma insulin-like growth factor-binding protein (IGFBP)-1 and 9 (Supplementary Figure 2). All DIO mice had severe liver steatosis (> 66% fat in the liver tissues) along with extensive collagen accumulation (Supplementary Figure 3).

After subcutaneous implantation of syngeneic ER^+^ E0771 BC cells, tumor growth increased more rapidly in DIO mice than in the control. Consistent with previous studies [28, 29], the tumor volume in obese mice was about 4 times greater than in CTL, and tumor weight was about three times greater after 21 days (Figure 1B and 1C; *p < 0.0001 and 0.05*). The expression levels of Ki67 (a cellular marker for proliferation) in the tumor tissues were also about four times higher in the DIO mice than in the controls (Figure 1D; *p < 0.01*).

Histomorphological analysis revealed that tumors from both groups had features of an aggressive and malignant phenotype, characterized by sheets of cells with moderate amounts of cytoplasm, marked nuclear pleomorphism, prominent nucleoli and brisk mitotic activity. Most tumors had extensive necrosis with an expansile growth pattern with pushing borders. Among 14 DIO mice, we observed a focally infiltrative growth pattern in one animal, and liver cancer in another. More tumors in DIO mice had a partial fibrous capsule compared to the lean control (data not shown).

### Diet-induced obesity stimulates microvascular remodeling via LPA/PKD-1-CD36 signaling axis

**Figure 2:**
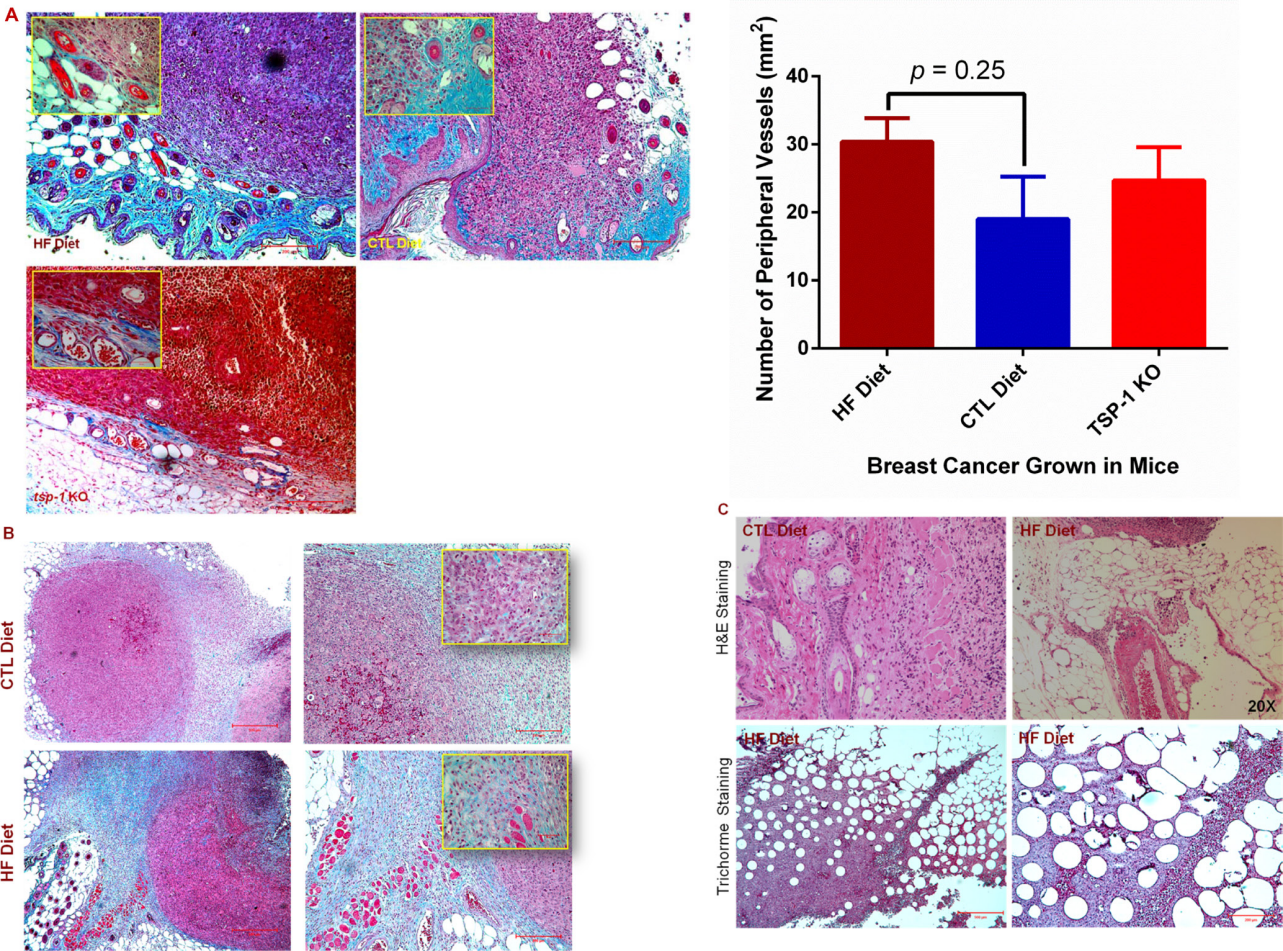
Diet-induced obesity promotes tumor angiogenic and metastatic potential (**A**) Paraffin-embedded tumor tissues were sectioned and stained by Trichrome. Inserts are magnified images of areas containing blood vessels (left panel). The number of blood vessels in the periphery of each tumor was quantified under a microscope, and the average number was compared in the tumor tissues between mice fed with HF and CTL diets (right panel). Bar = 200 μm in representative images or 50 μm in inserted images. (**B**) Tumors grown in DIO mice showed increased collagen accumulation in tumor-associated stroma as shown by blue/green color in the Trichrome staining. Inserts are magnified images, and bar = 200 μm in representative images. (**C**) H & E and Trichrome stain showed BC invasion into the fat tissues in DIO mice. Representative color images of H & E (upper panel, 20 X ) or Trichrome (lower panel) stained sections show many fat vacuoles in the BC tissues in the DIO mice, bar = 500 μm (lower left panel) and 200 μm (lower right panel).

Tumor angiogenesis is important in promoting neoplastic progression of tumors [10]. We thus assessed neovessels within tumor tissues, and found that the average number of peripheral blood vessels per tumor was about 40% higher in DIO mice compared with the lean control, but this difference was not statistically significant (*p = 0.25)*. Interestingly, this vessel density was comparable to that seen within the TME in *tsp-1* (thrombospondin-1) knockout mice, which had deficient expression of TSP-1 (a ligand of CD36) and showed robust tumor angiogenesis [30, 31]. While there was no significant difference in the vascular density in the tumors grown in the DIO mice when compared to the CTL (Figure 2A), we observed that excessive collagen was accumulated within the tumor extracellular matrix (Figure 2A and 2B). More strikingly, tumors invaded nearby fatty tissues in the DIO mice, and many fat vacuoles occurred within the tumors (Figure 2C). These results suggest that inflammatory and other proangiogenic processes may promote BC progression, in which factors produced by the fatty tissues may play an important role.

**Figure 3:**
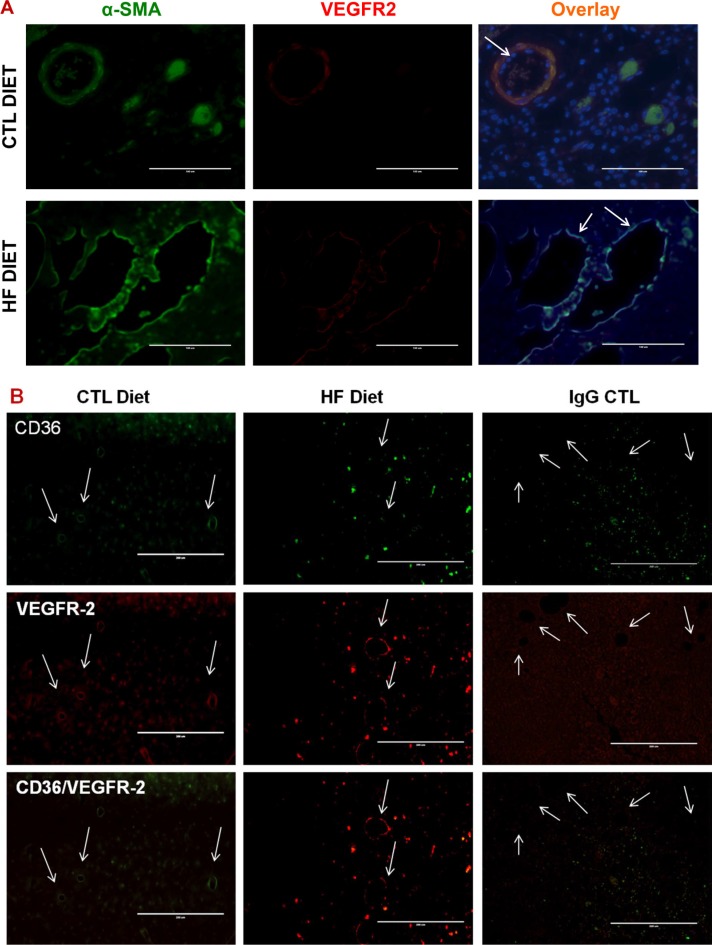

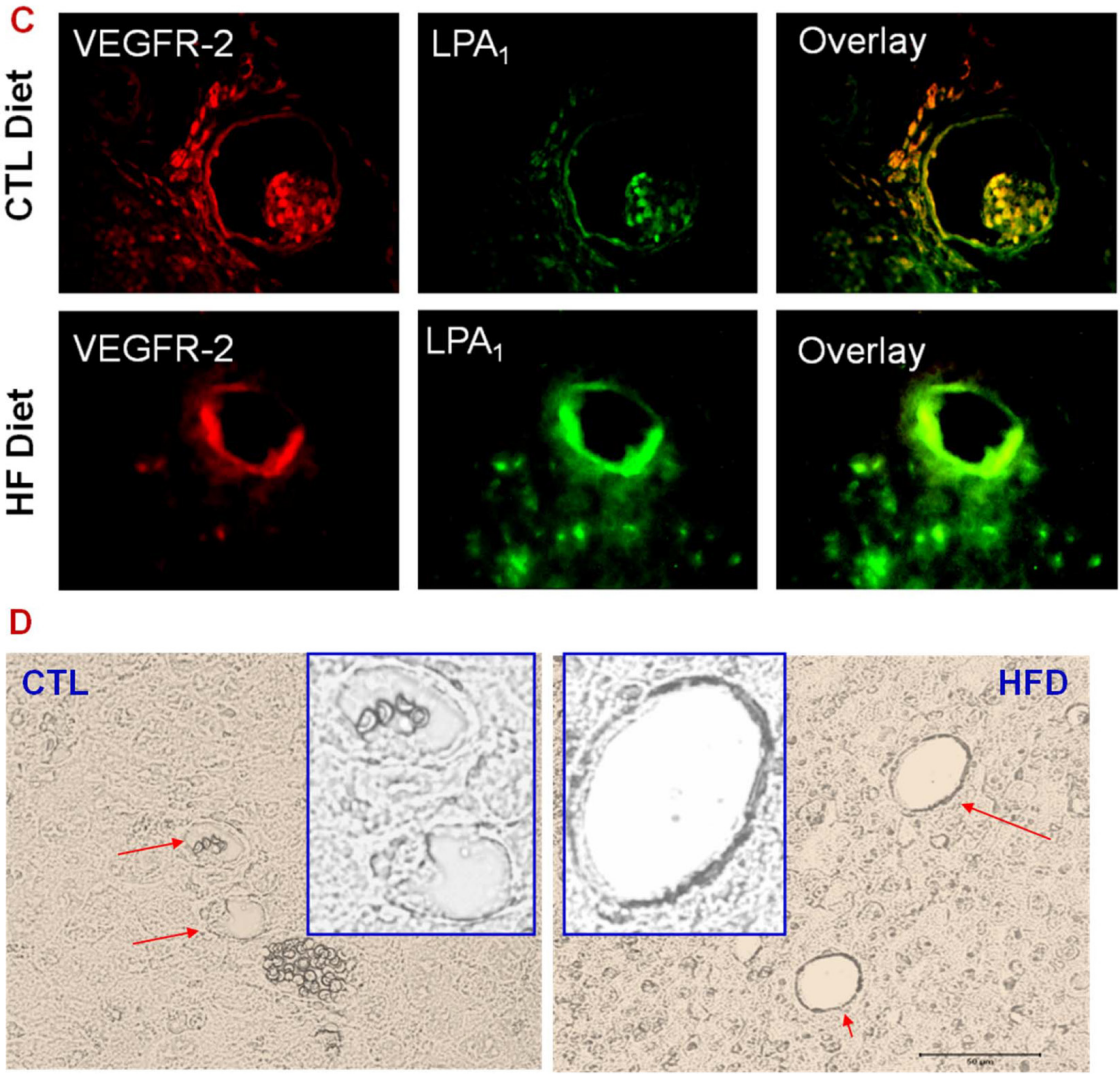
LPA/PKD-1 signaling in arteriogenic gene expression (**A**) Tumor sections were co-stained with anti-α-SMA antibody and anti-VEGFR2 antibody (e-Bioscience) followed by FITC (Santa Cruz) or biotin-labeled CYR3 secondary antibody and mounted with DAPI. Images were acquired and overlayed using an EVOS^®^FL cell imaging system. Bar = 100 μm in representative images. (**B**) Tumor sections were stained with anti-CD36, or anti-VEGFR2 antibodies as described in A. Images were acquired and overlayed using an EVOS^®^FL cell imaging system. Bar = 200 μm in representative images. Note: the obvious green staining of cells within the ER^+^ tumor tissues appeared to be CD36 positive cancer cells. (**C**) G-protein coupled receptor LPA_1_ expression increased in the tumor endothelium in DIO mice. Tumor sections were stained with anti-VEGFR2 and anti-LPA_1_ antibodies (Cayman Chemical) followed by appropriate secondary antibodies. Images were acquired as in Figure 1. (**D**) Phospho-PKD-1 levels increased in tumor vessels in DIO mice. Tumor sections were stained with phospho-PKD^Ser744-748^ antibodies and assessed using the VECTASTAIN^®^ABC with an alkaline phosphatase enzyme detection system. Images were acquired as in Figure 1D. Representative images are shown, in which vascular staining of phosphorylated PKD-1 within tissues are denoted by arrows (bar = 50 μm), and inserts are modified magnified images with arbitrary increase of contrast and magnification.

Obesity generates excessive LPA [25, 26], which promotes BC progression [11] and induces angiogenesis in a variety of conditions [12, 15–17, 32, 33] via specific G-protein coupled LPA receptors including LPA_1_ and possibly LPA_3_ [16, 34]. We previously showed that LPA regulated vascular remodeling and was implicated in capillary arterialization and *de novo* arteriogenesis [15, 19]. Arterial remodeling within tumors may promote cancer growth by formation of an arteriolar vasculature [35, 36]. To determine the properties of vascularization in BCs grown in DIO mice, we performed immunofluorescence microscopy. We observed that the expression levels of both vascular endothelial growth factor receptor 2 (VEGFR 2), an important receptor for arterial differentiation of ECs, and alpha smooth muscle actin (aSMA), a smooth muscle cell marker, were elevated, but CD36 expression levels were reduced in certain tumor vessels (Figure 3A and 3B) although similar levels of CD36 and VEGFR2 were present in other tumor vessels in the DIO mice (Supplementary Figure 4). Intriguingly, CD36 also appeared to express in a few tumor cells in DIO mice (Figure 3B).

To examine whether LPA signaling existed in the tumor vessels, we examined the expression of LPA receptors LPA_1_ and LPA_3_. We observed that the tumor endothelium showed noticeable LPA_1_ expression in lean mice, but the levels of expression increased in the DIO mice (Figure 3C). However, we did not find significant differences in the expression of LPA_3_ (data not shown). Since we had shown that PKD-1 was involved in downstream signaling of the LPA pathway in MVECs [15, 16], we examined the phosphorylation status of endothelial PKD-1, a downstream signaling of LPA pathway in the tumor endothelium. Indeed, the phosphorylation levels of PKD-1 were increased in the tumor endothelium in chronic DIO mice (Figure 3D).

### Sustained LPA/PKD-1 signaling in tumor associated endothelial cells is required for the regulation of endothelial differentiation

**Figure 4:**
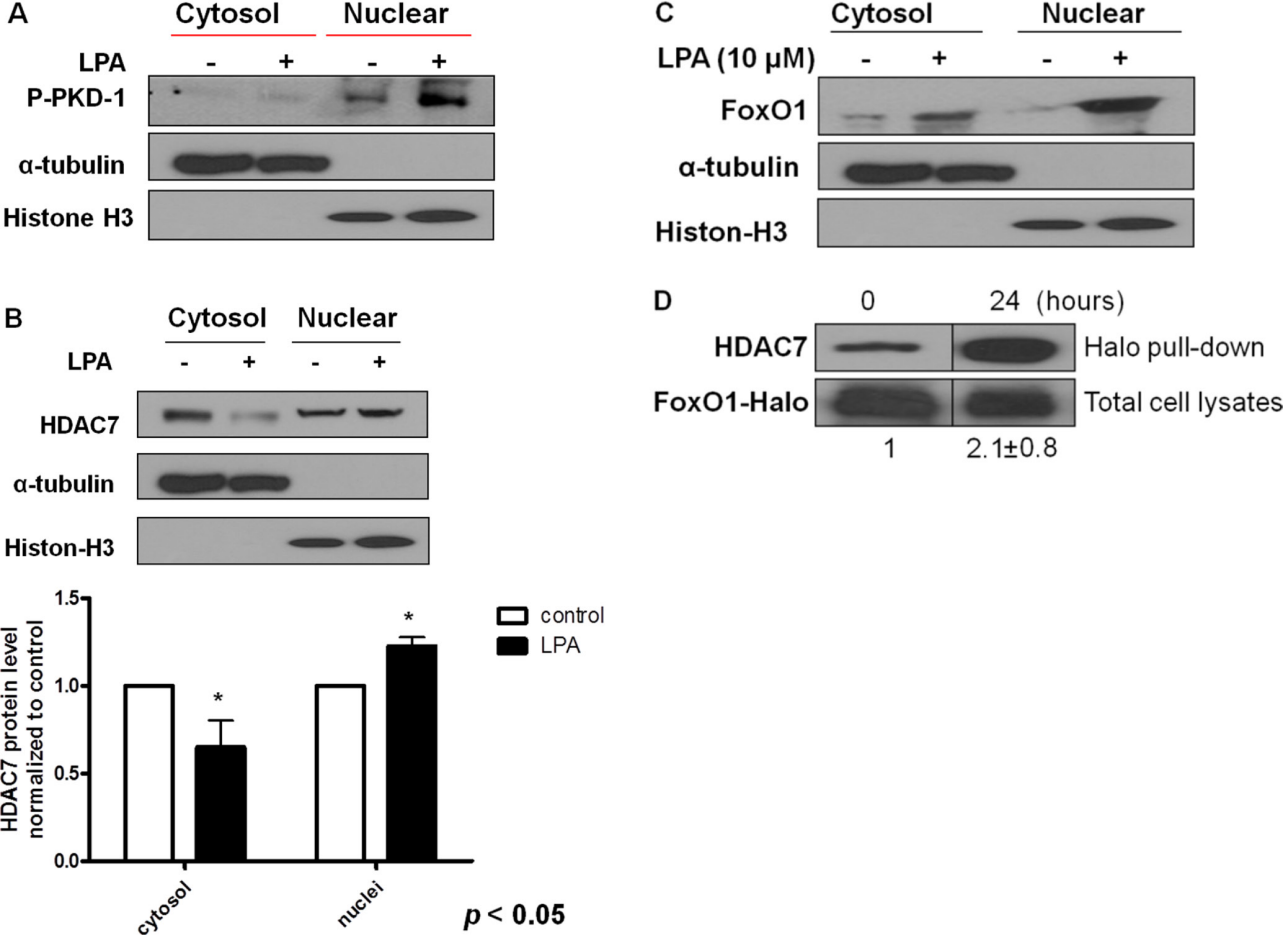
Sustained LPA/PKD-1 signaling may be involved in TAEC differentiation (**A**) TAECs were exposed to LPA (10 μM) for 24 hours. Cytosol and nuclear fraction were isolated, and phospho-PKD-1 ^Ser744-748^ levels were detected by Western blots. α-tubulin and histone 3 respectively represent cytosol and nuclear components. Representative images from three separate experiments are shown. (**B**) TAECs were treated as described in A, and cytosol and nuclear fractions were isolated for the detection of HDAC7. Representative images and quantified densitometry data from three separate experiments are shown. (**C**) TAECs were treated with LPA (10 μM), and cytosol and nuclear fractions were isolated for the detection of FoxO1 as indicated in A. Representative images from three separate experiments are shown. (**D**) TAECs were transfected with plasmids expressing Halo-tagged FoxO1 for 24 hours followed with LPA (10 μM) for an additional 24 hours for Halo pull-down assays.

LPA regulates *CD36* transcription through activating PKD-1 signaling in MVECs, which is important in arteriolar differentiation and arterial remodeling of microvasculature [15, 16, 20]. Endothelial cells are heterogeneous [37, 38] and TAECs are genetically unstable [39]. To determine the effect of LPA on the tumor endothelium and elucidate the mechanisms *in vitro*, we treated TAECs with LPA. Similar to our previous studies [16] and current *in vivo* studies, prolonged exposure of TAECs to LPA significantly increased the levels of phosphorylated PKD-1 in the nuclei (Figure 4A). Since histone deacetylase HDAC7 as a PKD-1 substrate and transcription factor FoxO1 regulated LPA/PKD-1 signaling-mediated CD36 transcription [15, 16], we examined nuclear localization of HDAC7 and FoxO1, and observed that both HDAC7 and FoxO1 were accumulated in the nuclei in response to LPA (Figure 4B and 4C), accompanying the increased molecular interactions between these two factors as shown by Halo-pulldown assays (Figure 4D).

**Figure 5:**
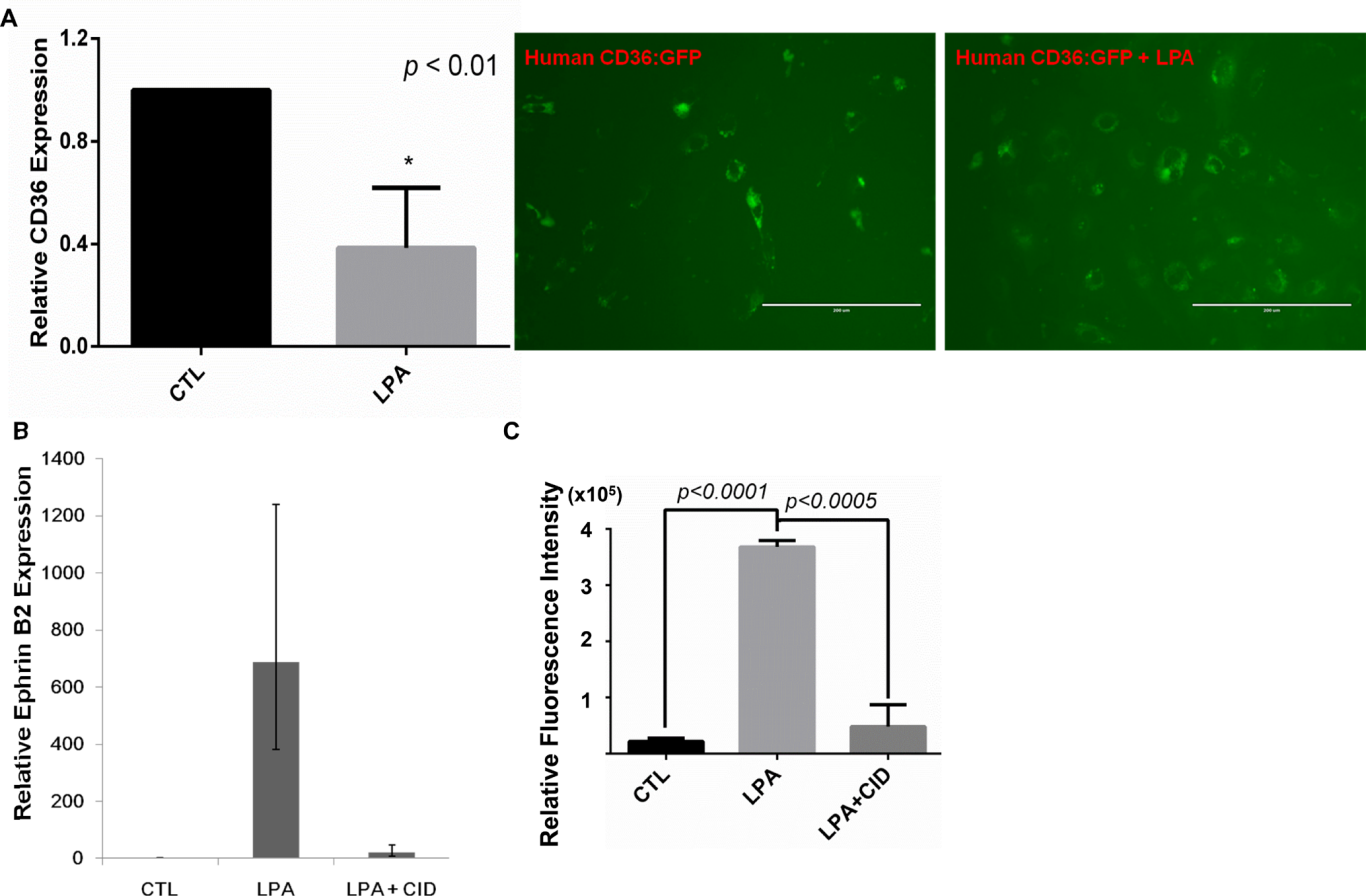
LPA/PKD-1 signaling in the expression of CD36 and ephrin B2 and endothelial cell proliferation (**A**) TAECs were treated with vehicle or LPA (10 μM) for 24 hours, and CD36 mRNA levels were assayed by real time qPCR (left panel); Cardiac ECs starved for 6 hours, were infected by lentivirus human CD36:GFP for 72 hours, with addition of LPA (10 uM) at the last 24 hours. Images were acquired using an EVOS^®^FL cell imaging system. (**B**) TAECs were exposed to LPA (10 μM) or LPA (10 μM) and PKD inhibitor CID 755673 (25 μM) for 48 hours, and total RNA was extracted for real time qPCR. A representative result is shown. (**C**) TAECs transduced with GFP were exposed to LPA (10 μM) or LPA (10 μM) and CID755673 (25 μM) for 24 hours for proliferation assays.

MVECs reprogram to express proangiogenic and proarteriogenic genes once CD36 expression is downregulated via LPA/PKD-1 signaling [15], which is essential in arterial differentiation [20]. To determine whether similar reprogramming processes in response to this signaling pathway occurred in tumor microvasculature, we treated TAECs with LPA with/without a selective PKD inhibitor and assessed the expression of key arteriogenic gene expression. To support our finding of CD36 expression in tumor endothelium *in vivo*, we first studied the expression of this molecule in TAECs in response to LPA treatment. We observed that CD36 mRNA levels were significantly reduced in TAECs exposed to LPA compared to the vehicle treated cells (Figure 5A left panel, *p* < 0.01). Moreover, LPA exposure suppressed the protein expression of CD36 in human cardiac ECs overexpressing CD36 (Figure 5A right panel). In contrast, LPA exposure increased the mRNA levels of ephrin B2 in TAECs, and this increase was associated with PKD activities (Figure 5B). Functionally, the LPA/PKD-1 signaling promoted TAEC proliferation (Figure 5C).

## DISCUSSION

Obesity increases BC risk, with multiple factors involved such as a primary metabolite of cholesterol or 27-hydroxycholesterol [28] and changes in the levels of adipocyte-derived factors [27, 40]. In this study, we highlight the importance of excessive LPA produced by chronic DIO in promoting BC progression. LPA/PKD-1-CD36 signaling-mediated microvascular remodeling may link diet-induced obesity to ER-positive breast cancer progression under chronic obese conditions. Stimulating microvascular remodeling that may lead to *de novo* tumor arteriogenesis rather than increasing microvascular density [15, 35, 36] could play a key role in the malignant progression of BCs. As previously reported [27, 40, 41], we observed much faster growth of ER^+^ BC in chronic DIO mice than in the lean control though we did not observe significant changes in vascular density within tumors. Interestingly, more collagen was accumulated within tumor tissues in the DIO mice, which may promote mammary tumor initiation and malignant progression [42]. These results suggest more inflammation and metastatic potential in BCs under chronic obese conditions.

Additionally, we observed that LPA/PKD-1 signaling is activated in the tumor endothelium possibly via the G-protein coupled receptor LPA receptor 1 (LPA_1_) [16]. Similar to our previous report [15], this pathway downregulates CD36 expression and promotes arteriolar remodeling in the TME, an essential process for enhancing tumor tissue perfusion and nutrition [43]. It is known that the expression of autotaxin (a key enzyme for LPA production) or LPA receptor in mammary epithelium of transgenic mice is sufficient to induce a high frequency of late-onset, invasive, and metastatic ER^+^ mammary cancer [11]. We also observed that direct LPA administration appeared to promote BC metastasis in a syngeneic model (data not shown). It is tempting to speculate that LPA/PKD-1-CD36 signaling-mediated vascular remodeling may facilitate the survival of circulating tumor cells in circulation due to their exposure to adipocyte- and tumor-released cytokines and growth factors by creating a favorable microenvironment.

CD36 is significantly repressed in multiple cell types of disease-free stroma associated with high mammographic density and tumor stroma [44]. CD36 downregulation in the neovessels may contribute to both inflammatory and microvascular remodeling processes under chronic obese conditions [11, 12, 15, 19]. On the other hand, CD36 can express in cancer stem cells or tumor initiating cells (TICs), promoting glioblastoma progression [22] or metastasis of breast, oral, and skin cancers [23]. Interestingly, our studies showed that CD36 appeared to express in certain BC cells in the DIO mice. Since LPA directly promotes BC progression [11], it is tempting to further study whether these cells are TICs and whether obesity-derived LPA drives CD36 expression in a specific subpopulation of cells and render them become metastatic TICs. In our current BC model in DIO, BC cells grew too fast and we had to sacrifice the mice before metastasis can be observed clearly. The model needs to be optimized to further investigate whether DIO promotes BC metastasis via both promoting arteriolar remodeling of microvasculature and development of metastatic TICs as well as precise mechanisms [23].

Furthermore, the increase of the G-protein coupled receptor LPA_1_ in the tumor endothelium may render TAECs more sensitive to DIO-derived LPA [26, 45], thereby leading to sustained nuclear PKD-1 signaling for *de novo* tumor arteriogenesis [15]. The direct link of this receptor in BC progression via microvascular remodeling needs to be explored by establishing an endothelial specific LPA_1_ knockout mouse line. However, it is reasonable to conclude that the proarteriogenic responses may be realized by LPA/PKD-1-CD36 signaling-mediated expression of ephrin B2, a key arterial marker and molecular signature in angiogenesis and arteriogenesis [46, 47].

Similar to previous studies in breast and other cancer types [15, 35, 40, 48], we observed enhanced vascular expression of aSMA and VEGFR2 along with CD36 downregulation in certain tumor vessels. This suggests that there is an increased investment of smooth muscle cells and/or pericytes in the tumor microvasculature. This mural cell investment and coverage is increased but appear insufficient in promoting complete vascular maturation and formation of functional arterioles. The interactions between FoxO1 and HDAC7, and altered FoxO1 activities [15, 49, 50] may also play an important role in these processes. The end point is increased expression of ephrin B2 and enhanced *de novo* tumor arteriogenesis [15, 46] for tumor progression.

The proarteriogenic process is also supported by increased VEGFR 2 expression in the tumor vessels under obesity conditions, which may result from increased leptin expression due to DIO [51]. High levels of VEGFR 2 expression may contribute to a change in TSP-1 initiated CD36 signaling in different types of cancer including breast, brain, colon, lung, and skin cancer [15, 30, 31, 44, 52–57]. Whereas CD36 downregulation in the tumor endothelium is associated with malignant lesions of BC [44], which may partially explain the role of TSP-1 as a CD36 ligand in BC metastasis [52]. It is intriguing that we observed that the LPA/PKD-1 signaling downregulated CD36 expression in TAECs, accompanying an increase in the expression of ephrin B2 and VEGFR 2 and TAEC proliferation. Additional studies are required to characterize how the LPA/PKD-1-CD36 signaling axis interacts with VEGFR 2 for ephrin B2 expression and microvascular remodeling-mediated BC progression.

**Figure 6:**
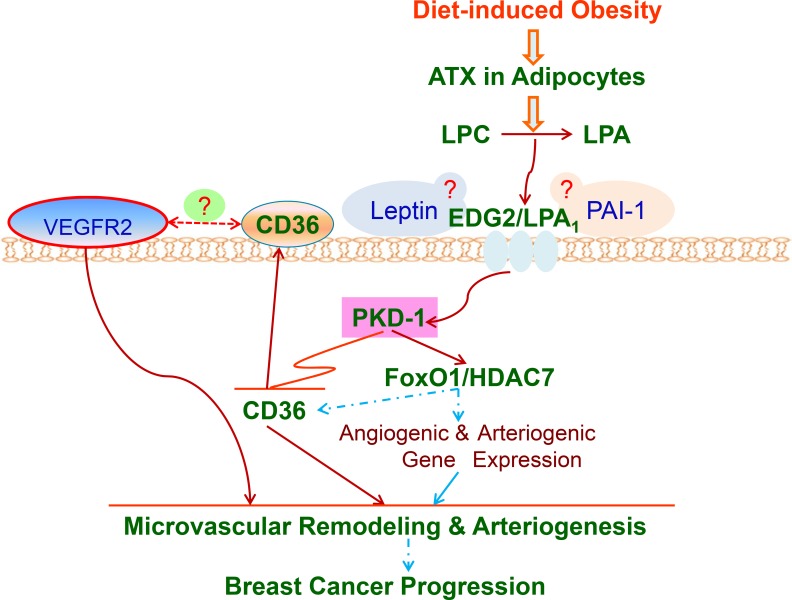
LPA/PKD-1-CD36 signaling axis in *de novo* tumor arteriogenesis in obesity A working model that shows the mechanisms by which LPA/PKD-1-CD36 signaling axis promotes arteriolar remodeling of microvasculature in BC for malignant progression via G-protein coupled receptor LPA_1_ in chronic DIO. ATX: autotaxin; LPC: lysophosphatidylcholine. Dash line means that mechanisms are unclear.

As DIO may generate many angiogenic factors in circulation, we performed proteome profiling of angiogenic regulators. Intriguingly, we observed that only levels of leptin and PAI-1 were elevated in the plasma of DIO mice among 53 angiogenesis associated proteins. It is known that both leptin and PAI-1 regulate angiogenesis [8, 58]. Leptin signaling also promotes the growth of mammary tumors and increases the expression of VEGFR 2 [5, 51]. Therefore, under chronic obese conditions a functional crosstalk may exist between LPA, leptin, and PAI-1 in the regulation of arteriolar remodeling of tumor microvasculature to promote *de novo* tumor arteriogenesis. Furthermore, changes in the levels of CD36 and VEGFR 2 expression may change the crosstalk between CD36 and VEGFR2 [14] and contribute to proarteriogenic processes for BC progression (Figure 6). The *de novo* tumor arteriogenesis may be accomplished through integrating proinflammatory and arteriogenic signals as well as by changing mitochondrial functions of TAECs and immune cell functions [41, 59, 60]. Obesity-derived LPA, leptin, and PAI-1 are all known as a promoter of BC progression [11, 61]. Our studies thus provide evidence for the concept that chronic obesity promotes tumor progression through non-estrogenic mechanisms [62].

Taken together, it is conceivable that the LPA/PKD-1-CD36 signaling axis under chronic obese conditions contributes to arteriolar remodeling of tumor microvasculature, leading to BC progression. CD36 downregulation in the tumor endothelium could be a key step that initiates the microvascular remodeling for *de novo* tumor arteriogenesis. Our studies also reveal a previously unknown molecular signaling pathway that promotes the microvascular remodeling in tumors, and may link obesity to tumor progression. This represents a point of convergence for microenvironmental signals in obesity to regulate a specific proarteriogenic process for malignant progression. This also demonstrates significant therapeutic implications because the microvascular remodeling that forms abnormal dilated and tortuous feeder arterioles later in tumor development may contribute to metastasis and the resistance to some antiangiogenic treatments [35, 63, 64].

## MATERIALS AND METHODS

### Cell lines

The ER^+^ E0771 mouse breast adenocarcinoma cell line was provided by Dr. Kamalakannan Rajasekaran (Blood Center of Wisconsin). Cells were maintained in RPMI 1640 supplemented with 10% fetal bovine serum and 10 mM HEPES. Tumor-associated endothelial cells (TAECs, 2H11) [65] were purchased from American Type Culture Collection (ATCC), and grown in DMEM supplemented with 5% fetal bovine serum.

### Tumor angiogenesis model

Animal experiments were approved by the Institutional Animal Care and Use Committee of the Medical College of Wisconsin. Six-week-old female C57BL/6 mice were purchased from Jackson Laboratory, and maintained on a high fat diet (HF diet, D12492, 60 kcal% fat, Research Diets, Inc., NJ) or a control chow diet (D12450B, 10 kcal% fat, Research Diets, Inc) for 32 weeks. Body weight was measured weekly. Only female mice were used in these studies because this tumor line is ER^+^, and this type of tumor is much less common among males than females. E0771 cells (1×10^6^ cells/mouse) were implanted into mice close to the fourth mammary pad [66] and tumor volume was measured using calipers in two dimensions and calculated using the formula: (width^2^ × length)/2 [31].

### Immunofluorescence and immunohistochemical assays

Tissue immunohistochemical and immunofluorescence staining was performed using antibodies and methods as described in our previous studies [15, 16, 47] or by using the Vectastain^®^ ABC kit (Vector Laboratories).

### Mouse angiogenesis profiling

A Proteome Profiler™ antibody array (R&D system^®^) was performed to determine relative protein levels of angiogenic factors in the plasma according to the manufacturer's instruction.

### Immunoblot and Halo pull-down assays

TAECs were treated with 1-oleoyl-LPA (Avanti Polar Lipids and Sigma-Aldrich), and lysates were collected and subjected to cytosol/nuclei fractionation by a nuclei extraction kit (Millipore). Western blots were used for the detection of targeted proteins with appropriate antibodies as previously described [15]. Bands on immunoblots were quantified using NIH Image J software. For transfection and Halo pull-down assays, cells were transfected with the plasmids expressing Halo-tagged FoxO1 (Molecular Biology Core, Blood Center of Wisconsin) using Lipofectamine 2000 (Invitrogen) for 24 hours. Transfected cells were treated with LPA (10 μM) for an additional 24 hours before cell lysates were collected and subjected to the Halo pull-down assays according to the manufacturer's instruction (Promega).

### Real time qPCR

Gene expression was assessed by real-time qPCR as previously described [31]. Relative quantitation experiments were designed and performed in an ABI 7500 or QuantStudio Real-Time system (Life Technologies^®^). RT2 qPCR primer assays (QIAGEN) for the target genes and housekeeping genes were used for PCR reactions.

### Transduction of CD36 into human cardiac endothelial cells

Human CD36 plasmid pLCP-hCD36:GFP-Puro was constructed by cloning hCD36 into pLenti-CMV-GFP-Puro (Addgene plasmid 17448) using InFusion (Clontech), and used with psPAX2 (Addgene plasmid 12260) and pMD2G (Addgene plasmid 12259) to produce Lentivirus by PEI transfection of 293T cells. Primary (Blood Center of Wisconsin) cardiac ECs were infected with the lentivirus for the overexpression of CD36.

### Proliferation assays

TAECs transduced with GFP were seeded into 48 well plates, and cellular proliferation was quantified using the almarBlue^®^ Cell Viability assay kit (invitrogen™) with an EnSpire^®^ multimode plate reader (PerkinElmer) at excitation and emission wavelengths of 570 nm and 585nm. Two independent experiments were carried out, and the data were expressed as the mean ± SD of assays performed in triplicate wells.

### Statistics

Quantitative data are presented as mean ± SD or SEM. Comparisons were done by Student *t* tests. A *p* < 0.05 was considered statistically significant.

## SUPPLEMENTARY FIGURES


