# Supplementary material for: Diet-induced obesity links to ER positive breast cancer progression via LPA/PKD-1-CD36 signaling-mediated microvascular remodeling
Source: Oncotarget. 2017 Feb 6;8(14):22550–62. doi: 10.18632/oncotarget.15123 (PMC5410244; doi:10.18632/oncotarget.15123)
Supplement: Supplementary file 1 [file oncotarget-08-22550-s001.pdf]

Diet-induced obesity links to ER positive breast cancer progression via LPA/PKD-1-CD36 signaling-mediated microvascular remodeling

Supplementary Materials

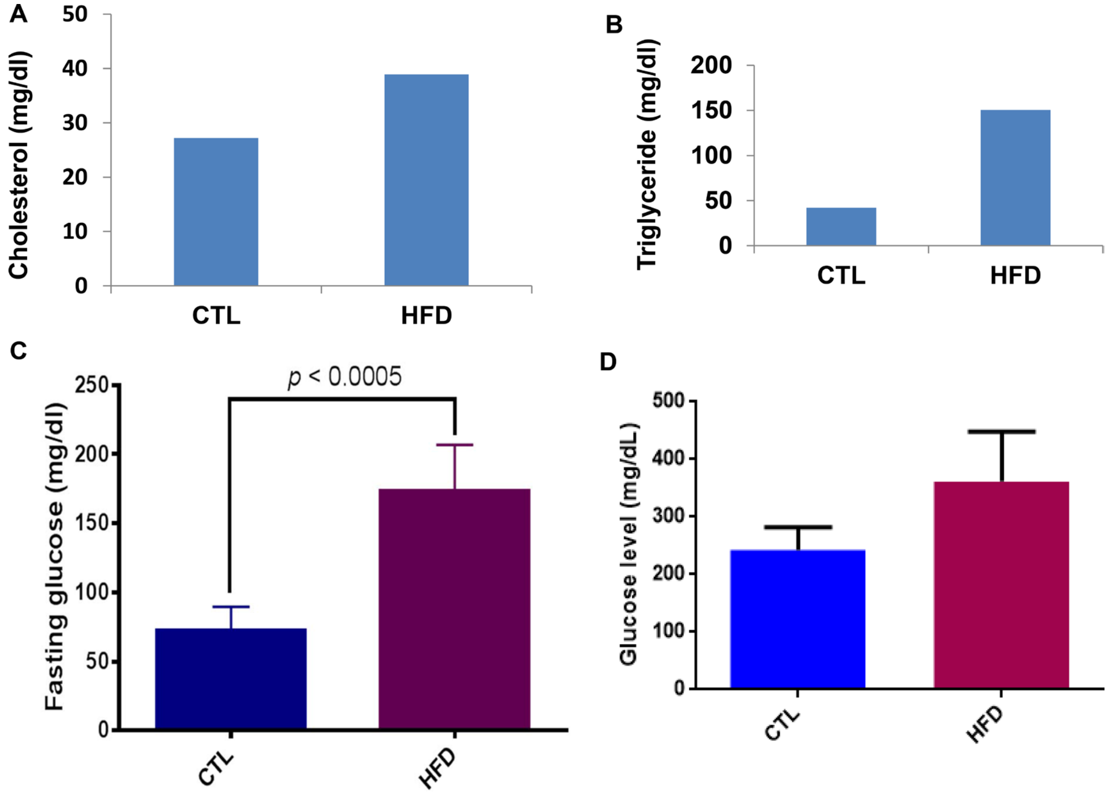

Supplementary Figure 1: (**A**) Plasma cholesterol was measured by the Amplex Red cholesterol assay kit (Invitrogen). All assays were conducted according to the manufacturer's instruction. (**B**) Plasma triglycerides was measured by the triglyceride assay kit (Cayman Chemical). All assays were conducted according to the manufacturer's instruction. (**C**) The glucose level increased in DIO mice by overnight fasting when compared with the lean control. Using a blood sugar meter, blood glucose was measured from tail vein blood in mice with fasting after they were fed with a control or high fat diet for 32 weeks ($p < 0.0005$). (**D**) Blood glucose levels did not change without overnight fasting in the DIO mice when compared to the lean control ($p > 0.05$).

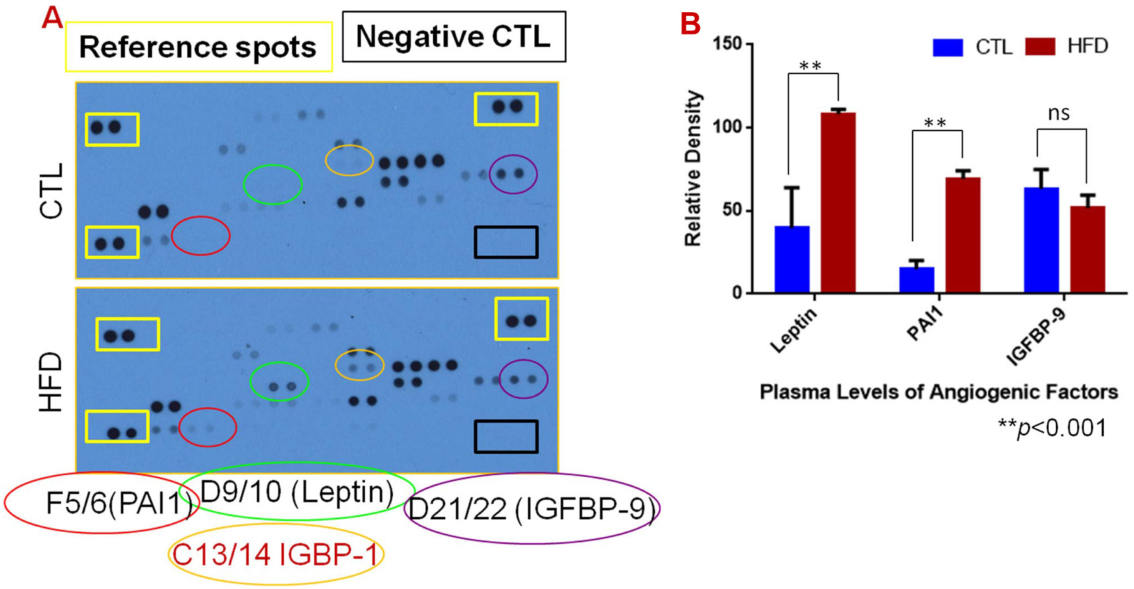

Supplementary Figure 2: The levels of leptin and PAI 1 were significantly increased in the plasma of DIO mice. The Proteome Profiler™ Mouse Angiogenesis kit was used to determine the relative expression of 53 angiogenic proteins in the plasma of diet-induced obese mice compared with the lean control. ImageQuant TL in ImageQuant LAS4000 (GE Healthcare Life Sciences) was used for imaging acquisition and array analysis to determine the signal density. The representative blots are shown in the left panel. The blotting dots with signal density that appeared to be different by the naked eye were analyzed, and the relative density is shown in the right panel. Note: The specific dots are indicated by circles of different colors. *C13/14 IGBP-1 does not show consistent results in two separate experiments.

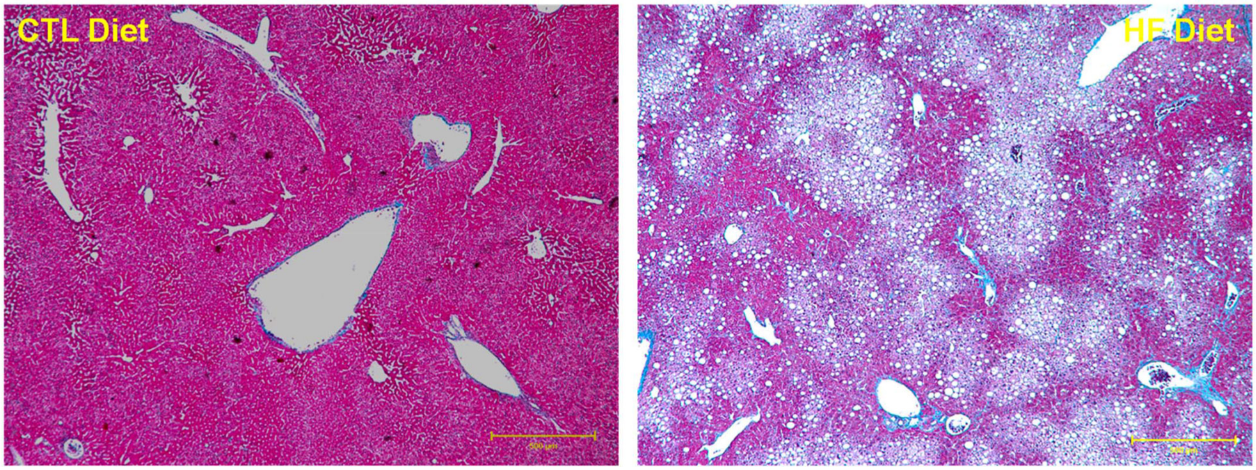

Supplementary Figure 3: Fat and collagen accumulation in the liver was shown by Trichrome staining in the DIO mice. Bar = 500 µM in representative images.

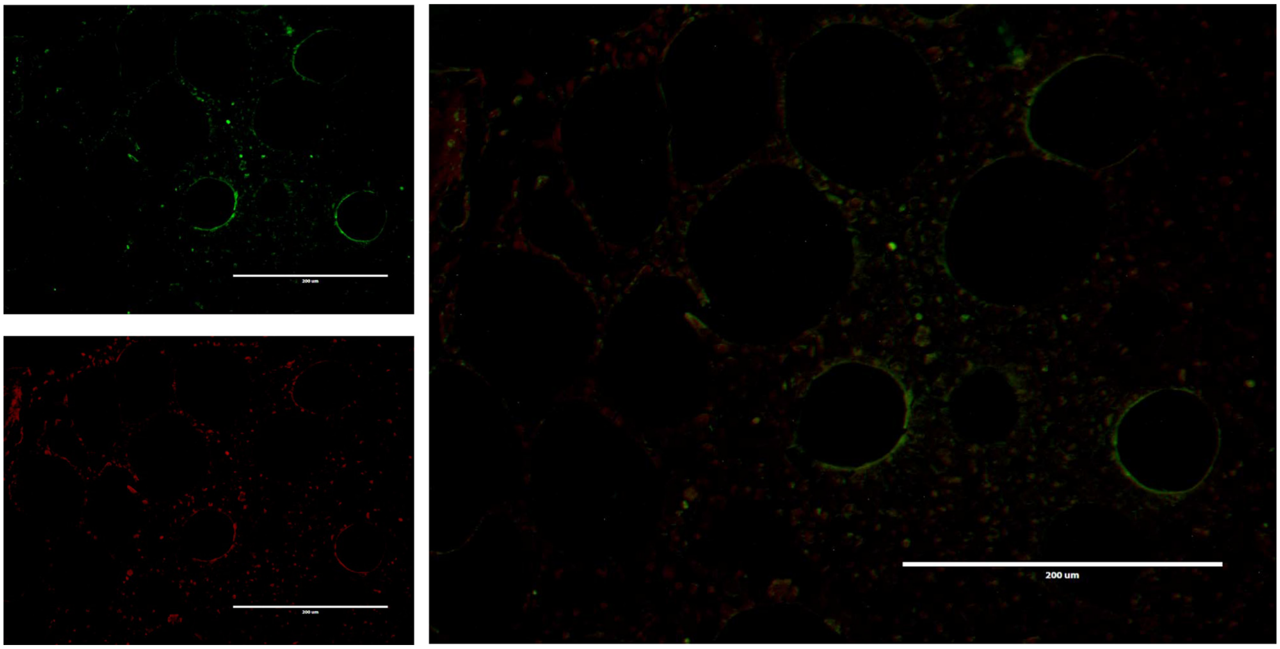

Supplementary Figure 4: Tumor vessels express both CD36 and VEGFR 2 in DIO mice. Tumor sections were stained with anti-CD36 or anti-VEGFR2 antibodies, and images were acquired as described in Figure 2A.
